# Study of diagnostic accuracy of Helmintex, Kato-Katz, and POC-CCA methods for diagnosing intestinal schistosomiasis in Candeal, a low intensity transmission area in northeastern Brazil

**DOI:** 10.1371/journal.pntd.0006274

**Published:** 2018-03-08

**Authors:** Catieli Gobetti Lindholz, Vivian Favero, Carolina de Marco Verissimo, Renata Russo Frasca Candido, Renata Perotto de Souza, Renata Rosa dos Santos, Alessandra Loureiro Morassutti, Helio Radke Bittencourt, Malcolm K. Jones, Timothy G. St. Pierre, Carlos Graeff-Teixeira

**Affiliations:** 1 Laboratório de Biologia Parasitária, School of Sciences, Pontifícia Universidade Católica do Rio Grande do Sul, Porto Alegre, Brazil; 2 School of Biological Sciences, Queen’s University Belfast, Belfast, Northern Ireland; 3 School of Physics, The University of Western Australia, Crawley, Western Australia, Australia; 4 Polytechnic School, Pontifícia Universidade Católica do Rio Grande do Sul, Porto Alegre, Brazil; 5 School of Veterinary Science, The University of Queensland, Queensland, Australia; Imperial College London, UNITED KINGDOM

## Abstract

Control initiatives have successfully reduced the prevalence and intensity of schistosomiasis transmission in several localities around the world. However, individuals that release low numbers of eggs in their feces may not be detected by classical methods that are limited by low sensitivity. Given that accurate estimates of prevalence are key to implementing planning control actions for the elimination of schistosomiasis, new diagnostic tools are needed to effectively monitor infections and confirm transmission interruption. The World Health Organization recommends the Kato-Katz (KK) thick smear as a parasitological test for epidemiological surveys, even though this method has been demonstrated to underestimate prevalence when egg burdens are low. The point-of-care immunodiagnostic for detecting schistosome cathodic circulating antigen (POC-CCA) method has been proposed as a more sensitive substitute for KK in prevalence estimations. An alternative diagnostic, the Helmintex (HTX) method, isolates eggs from fecal samples with the use of paramagnetic particles in a magnetic field. Here, a population-based study involving 461 individuals from Candeal, Sergipe State, Brazil, was conducted to evaluate these three methods comparatively by latent class analysis (LCA). The prevalence of schistosomiasis mansoni was determined to be 71% with POC-CCA, 40.% with HTX and 11% with KK. Most of the egg burdens of the individuals tested (70%) were < 1 epg, thereby revealing a dissociation between prevalence and intensity in this locality. Therefore, the present results confirm that the HTX method is a highly sensitive egg detection procedure and support its use as a reference method for diagnosing intestinal schistosomiasis and for comparative evaluation of other tests.

## Author summary

Schistosomiasis is a parasitic infection that is caused by flatworms that live inside venous vessels. The parasite eggs are eliminated with human feces. They hatch in the water and the free swimming larvae infect snails. The larvae develop in the snail tissues and, on reaching an infective stage, are released into the water and can penetrate human skin to restart the cycle. Millions of people on several continents have schistosomiasis, although sanitary improvements, treatment, and environmental control have reduced the intensity of transmission in various localities around the world. In order to eliminate schistosomiasis, identification of infected individuals is critical. However, in areas where transmission has declined, the methods used to detect eggs in human feces are not sufficiently sensitive. Therefore, the aim of the present study was to evaluate new and more sensitive methods for obtaining a diagnosis of schistosomiasis. This study compared the relative performance of the POC-CCA method, which detects parasite molecules released in urine and the Helmintex (HTX) method, which isolates eggs from large volumes of feces in a magnetic particle-based assay, together with the Kato-Katz method. In comparison with the egg detecting methods (Kato-Katz [KK] and HTX), the POC-CCA method exhibited limitations in detecting low intensity infections, while the HTX method was confirmed to be a highly sensitive diagnostic method for schistosomiasis. These results provide insights into the deployment of diagnostic tools for efforts to eliminate schistosomiasis in low endemic regions.

## Introduction

Schistosomiasis is a common infection that affects over 290 million individuals, especially in Sub-Saharan Africa, Asia, and South America [1]. In Brazil, the sole agent of schistosomiasis is *Schistosoma mansoni*, responsible for intestinal schistosomiasis. This species is endemic to northeastern and southeastern regions in Brazil, although focal transmission sites have been reported in other regions [2].

It is challenging to diagnose schistosomiasis in areas of low endemicity where prevalence and worm burden have decreased [3]. Classical diagnostic methods lack sensitivity in populations where effective control measures have reduced transmission or in areas where the parasite has recently been introduced [4,5]. Detection methods that employ antibodies [6], antigens [7], or DNA [8] have exhibited high sensitivity but reduced specificity compared to microscopy-based assays [9] and they are semi-quantitative [10]. The Kato-Katz (KK) fecal smear method [11] is recommended by the World Health Organization for routine use in epidemiological surveys as part of control measures in endemic areas [12]. The KK method has exhibited good performance in high endemic areas and is still applied in diagnostic surveys due to its ease of application and specificity. However, KK is not an accurate diagnostic in many situations, for example in situations where part of a population has been previously treated and low egg burden is present in stool [13]. In addition, because the volume of stool needed for the KK method is very small (< 50 mg), and eggs may be unevenly distributed in feces [14,15], a large fraction of true positives may be missed with the KK method [13].

To address these limitations, the Helmintex (HTX) method was developed [16] to specifically detect light infections. The HTX method is based on interactions between *S*. *mansoni* eggs and superparamagnetic particles in a magnetic field. Seeding experiments have demonstrated 100% sensitivity with this method for egg burdens higher than 1.3 epg [16]. Biophysical properties of the egg surface that may contribute to the performance of the HTX method have also been extensively studied [17,18]. Furthermore, Favero et al. [19] recently proposed an optimization of the HTX method that makes it less time consuming and more efficient for field surveys.

A point-of-care immunodiagnostic for detecting schistosome cathodic circulating antigen in urine (POC-CCA method) has been proposed as a substitute for the KK method based on its estimated higher sensitivity and operational advantages, especially in highly endemic areas. However, even with the advantages of the POC-CCA method, there is still a need for a highly sensitive and direct method for detecting eggs that can serve as a reference in performance evaluations. Other direct diagnostic methods, like biopsy, are not feasible for populational-based studies. Antigen detection methods, like POC-CCA, may indicate the presence of worms that are not excreting eggs at that time, but further studies are required for extensive evaluation of its specificity. Thus, the aim of the present study was to compare by latent class analysis the performances of the HTX, KK, and POC-CCA methods in an endemic area for schistosomiasis in northeastern Brazil and to evaluate the following hypotheses: i) highly sensitive methods can be evaluated in medium-high endemic areas rather than in low endemic areas if they include large numbers of low intensity infections and ii) the HTX method has the capacity to serve as a reference egg detection method due to its high sensitivity.

## Materials and methods

### Area of study and population

Between October and November 2015, a prospective community- and geographically-based study was conducted in the locality of Candeal, Municipality of Estancia, State of Sergipe, Northeastern Brazil (11° 16' 04" S 37° 26' 16" W). Approximately 700 people live in Candeal, a restricted area with houses in close proximity that is well delineated by a federal highway (BR-101) on its west side, a stream to the south, and farms on its northern and eastern borders. Schistosomiasis morbidity was not addressed. Routine yearly fecal convenience sampling and examination by KK (but with poor coverage) is followed by treatment provided by local health service.

### Ethical issues

A total of 580 individuals living in Candeal provided written informed consent to participate in this study. For the children and teenagers included in this study (aged 1–17 years), consent was provided by their parents or legal guardian. The protocol for this study was approved by the PUCRS Ethics Committee (register 48809715.1.0000.5336).

### Sample collection and examination protocols

Study design and reporting follow the Standards for the Reporting of Diagnostic accuracy (STARD-2015: http://www.stard-statement.org/) (S1 Checklist and S1 Flow Diagram).

### Fecal samples

Each individual received a large container (1 L) and was directed to collect an entire evacuation. Feces were processed immediately upon their arrival in the laboratory.

A commercial KK kit (HelmTest, Biomanguinhos, Brazil) was employed for the preparation of slides with fecal smears, according to the manufacturer's instructions. Briefly, each sample was placed on a paper, then a metal mesh was pressed over it. The sieved feces were then scraped into a plastic circular template mounted on a glass slide so that the template was filled. After removing the template, cellophane coverslips presoaked in a glycerin-malachite green solution were placed over the sieved feces. Two slides of each sample were prepared, labeled, and vertically stored in plastic boxes to ensure that no contact occurred between the slides. The boxes were kept in a refrigerator until they were examined by light microscopy. Each slide was completely screened by optical microscopy at a final magnification of 100× for the identification and quantitation of *S*. *mansoni* eggs. Egg per gram (epg-KK) values were calculated based on the average number of eggs counted on two slides, and multiplied by 24.

**Fig 1:**
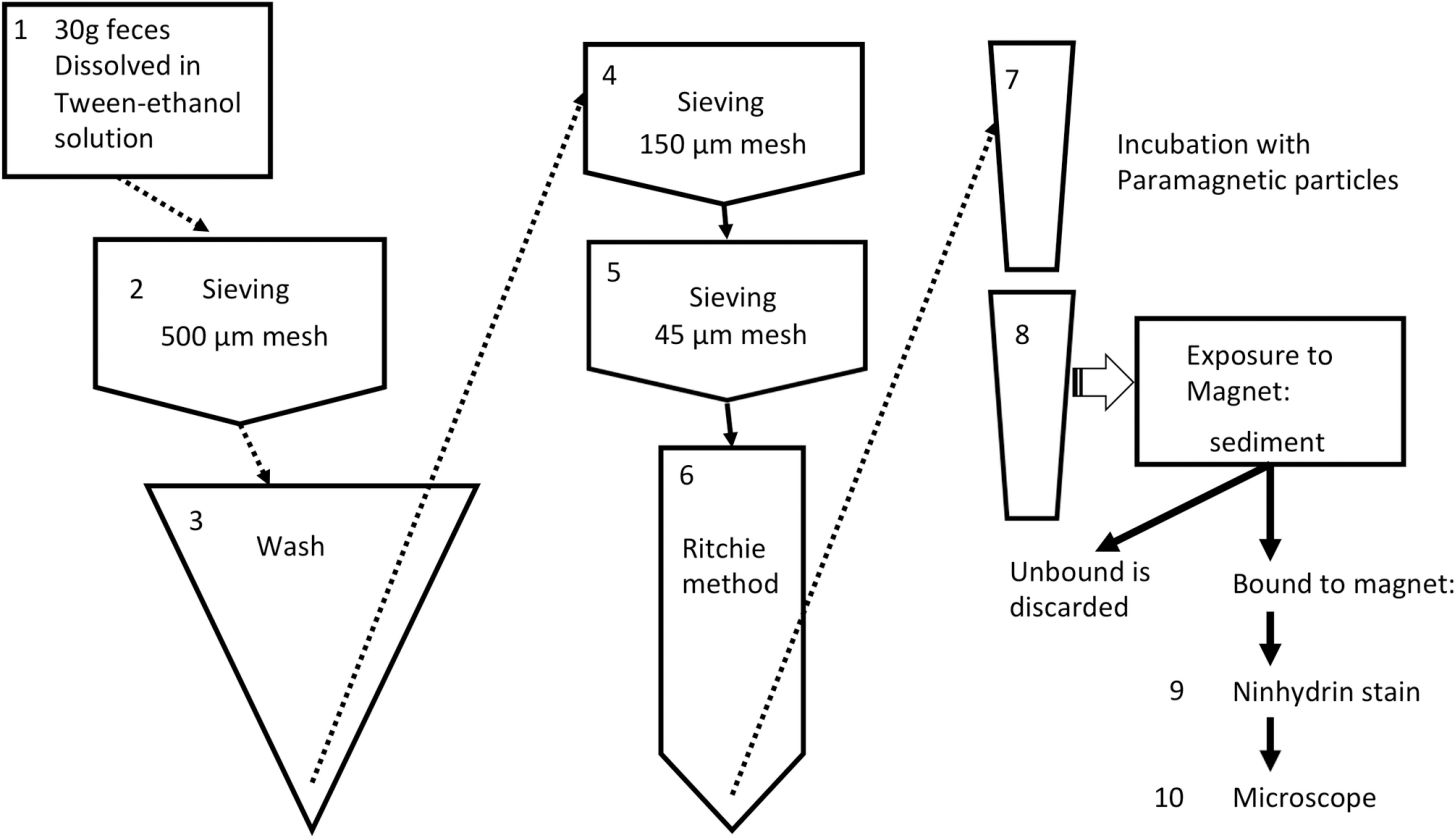
Schematic illustration of procedures in Helmintex method. It includes concentration steps from 1 to 8, followed by detection steps (9 and 10): ninhydrin staining and microscopic examination.

The HTX method was performed as previously described by Favero et al. [19] (Fig 1). Briefly, 30 g of feces was dissolved and fixed in a 10% Tween-20/70% ethanol solution (v/v). After 30 min, each suspension was passed through a 500 μm metal mesh, transferred to a conical flask, and washed until a clear supernatant was obtained. The suspension was then successively sieved through metal meshes with openings of 150 μm and 45 μm, respectively. The fraction retained by the last sieve was suspended in a 30% (v/v) ethyl acetate aqueous solution, homogenized and centrifuged for 10 min. at 200 x**g**. The pellet was collected after discarding the debris ring at the top of the aqueous phase (a modified Ritchie method) [20]. Each pellet was transferred to a microtube containing 19 μL of iron oxide paramagnetic particles (Bangs Labs, USA). After the pellets and particles were allowed to homogenize for 30 min with orbital rotation, the microtubes were placed in a magnetic rack (Bangs Labs, USA) for 3 min. Unbound material was discarded before each tube was removed from the rack. The magnetic-responsive pellets were then resuspended in 100 μL of 0.9% aqueous NaCl solution (w/v) and stored at -4°C until analyzed. To prepare the samples for microscopy analysis, each sediment was suspended and stained in 3% ninhydrin (Sigma-Aldrich, USA) in 70% ethanol (v/v) and homogenized by pipetting. Each suspension was evenly spread over 5 cm × 2.5 cm filter papers (24-μm pore) (UNIFIL, Brazil), identified, and kept for examination by optical microscopy (magnification, 100×). At the time of microscopy, the filters were moistened with drops of 70% ethanol (v/v) before the total number of eggs present were counted. The filters were stored separately in paper envelopes to avoid cross-contamination between samples. The sum of the eggs detected on all of the filter papers was divided by 30 to express the number of epg of feces. Throughout the text “epg” is meant to be the egg burden estimated by HTX.

### Urine samples

Each participant received a 200 mL container for urine collection. From each sample, 4 mL of urine was aliquoted and stored at -20°C for subsequent analyses.

POC-CCA tests were performed according to the manufacturer's instructions (Rapid Medical Diagnosis, Pretoria, South Africa). Briefly, a drop of urine was placed in a cassette and then a drop of developing reagent was added. Each cassette was kept at room temperature and the presence of a control line and a test line was checked exactly 20 min after the application of each sample. The results were recorded and independently verified by three trained observers. These results were also recorded by a digital camera under identical exposure settings. These images were reviewed by the same three observers for classification of intensity of test bands according to criteria (weak, medium, strong) proposed by Silveira et al. [21]. For most samples, the three sets of observations were in agreement. When different scores were recorded for a sample, the predicted infection status of the samples was reviewed by all three observers. If this review could not resolve the status, the final result was determined based on the observations of two of the three observers. “Trace” was considered a faint line with at least part of its limits not defined or absent. The instructions of the manufacturer states that “positivity” is any color that develops at the expected test site, but we register the result “trace” as defined above to allow a detailed comparative evaluation of the diagnostic methods, as previously reported by other authors [21, 22, 23].

### Blood samples

Venous blood was collected. Serum was stored at -20°C for transportation and then was stored at -80°C at the laboratory for future serological studies. No adverse effects were reported after collection of any biological samples.

### KK and HTX egg detection

The rationale for KK and HTX test positivity is that identification of an egg is pathognomonic for infection. Eggs of *S*.*mansoni* were identified using criteria as described by Favero et al. [19]. Performers and readers were blinded to other tests results or any clinical/epidemiological information.

### Statistical analysis

Assuming no diagnostic test is a “gold-standard”, relative diagnostic performance was accessed by latent class analysis (LCA) for each of the tests conducted based on sensitivity, specificity, positive predictive value (PPV), negative predictive value (NPV), and accuracy of the tests. Latent class models estimate prevalence and diagnostic accuracy based on the observed data from different tests. It is assumed that 2 latent classes correspond to groups of truly infected and non-infected individuals [24]. The 461 qualitative results from KK, POC-CCA and HTX methods were starting data points for LCA available in R software, Package e1071 (https://cran.r-project.org, assessed on November 4, 2017).

Classification as “false-positive” or “false-negative” was based mainly on the estimates from LCA but it should not be taken as definitive since in the present study neither irregular daily egg elimination in feces (false-negative results from egg-detecting tests, KK and HTX) nor laboratory cross-contamination (HTX false-positive results) could be ruled out.

The Fisher exact test was used to compare the proportions of positive results obtained from KK, HTX and POC-CCA. The Cohen’s Kappa coefficient was used to evaluate the agreement between the methods. The Student’s t-test, ANOVA and Tukey’s test were used to compare the means of epg in each category of POC-CCA band intensity.

## Results

### The HTX method is more sensitive than the KK method, while the POC-CCA method produced divergent results

A total of 681 individuals from Candeal, Brazil agreed to submit at least one type of biological sample for analysis in this study. A total of 461 (68%) participants donated feces, blood, and urine samples. Age groups comprised of 49 (10.7%) individuals less than 7 years-old, 92 (20%) school-aged-children (7 to 14 years-old) and 319 (69.3%) teenagers and adults. In order to compare all three methods, only results from those participants who provided all three specimens were examined (Tables 1–5 and S1 Table).

**Table 1:** A comparison of Kato-Katz (KK) and Helmintex (HTX) diagnostic methods that were applied to samples collected from the community of Candeal, Estância, Sergipe, northeastern Brazil, November 2015 (n = 461).

	HTX positive	(%)	HTX negative	(%)	Total	(%)
KK positive	54	(11.7)	1	(0.2)	55	(11.9)
KK negative	133	(28.9)	273	(59.2)	406	(88.1)
Total	187	(40.6)	274	(59.4)	461	(100.0)

**Table 2:** A comparison of point-of-care cathodic circulating antigen detection in urine (POC-CCA) and Kato-Katz (KK) diagnostic methods that were applied to samples collected from the community of Candeal, Estância, Sergipe, northeastern Brazil, November 2015 (n = 461). For this analysis, POC-CCA resulting in a faint line with at least part of its limits not defined or absent (“trace”) was considered a positive result.

	POC-CCA positive	(%)	POC-CCA negative	(%)	Total	(%)
KK positive	47	(10.2)	8	(1.7)	55	(11.9)
KK negative	283	(61.4)	123	(26.7)	406	(88.1)
Total	330	(71.6)	131	(28.4)	461	(100.0)

**Table 3:** A comparison of point-of-care cathodic circulating antigen detection in urine (POC-CCA) and Helmintex (HTX) diagnostic methods that were applied to samples collected from the community of Candeal, Estância, Sergipe, northeastern Brazil, November 2015 (n = 461). For this analysis, POC-CCA resulting in a faint line with at least part of its limits not defined or absent (“trace”) was considered a positive result.

	POC-CCA positive	(%)	POC-CCA negative	(%)	Total	(%)
HTX positive	153	(33.2)	34	(7.4)	187	(40.6)
HTX negative	177	(38.4)	97	(21.0)	274	(59.4)
Total	330	(71.6)	131	(28,4)	461	(100.0)

**Table 4:** A comparison of point-of-care cathodic circulating antigen detection in urine (POC-CCA) and Kato-Katz (KK) diagnostic methods that were applied to samples collected from the community of Candeal, Estância, Sergipe, northeastern Brazil, November 2015 (n = 461). For this analysis, POC-CCA resulting in a faint line with at least part of its limits not defined or absent (“trace”) was considered a negative result.

	POC-CCA positive	(%)	POC-CCA negative	(%)	Total	(%)
KK positive	40	(8.7)	15	(3.2)	55	(11.9)
KK negative	147	(31.9)	259	(56.2)	406	(88.1)
Total	187	(40.6)	274	(59.4)	461	(100.0)

**Table 5:** A comparison of point-of-care cathodic circulating antigen detection in urine (POC-CCA) and Helmintex (HTX) diagnostic methods that were applied to samples collected from the community of Candeal, Estância, Sergipe, northeastern Brazil, November 2015 (n = 461). For this analysis, POC-CCA resulting in a faint line with at least part of its limits not defined or absent (“trace”) was considered a negative result.

	POC-CCA positive	(%)	POC-CCA negative	(%)	Total	(%)
HTX positive	107	(23.2)	80	(17.3)	187	(40.6)
HTX negative	80	(17.3)	194	(42.1)	274	(59.4)
Total	187	(40.6)	274	(59.4)	461	(100.0)

The results obtained from analyzing the fecal samples with the HTX and KK methods, and analyzing the urine samples with the POC-CCA method, are compared in Tables 1–5. The prevalence estimates that were obtained varied according to the method used. For example, a total of 187 (40.6%) and 55 (11.9%) samples were positive for *S*. *mansoni* eggs according to the HTX and KK methods, respectively (S2 Table). When the samples were analyzed with the POC-CCA method, 330 (71.6%) and 187 (40.6%) samples were positive for schistosomiasis when trace results were considered positive rather than negative, respectively (Tables 2–5).

The relative diagnostic performance of the tests was estimated by LCA and the resulting values are presented in Table 6. The KK and HTX methods were in agreement regarding 54 (11.7%) positive samples and 273 (59.2%) negative samples. However, in 133 (28.9%) samples, schistosomiasis was only diagnosed by the HTX method (kappa coefficient = 0.329).

**Table 6:** Comparisons of the estimated sensitivity, specificity, positive predictive value (PPV), negative predictive value (NPV), and accuracy values after latent class analysis comparing Kato-Katz (KK) and, Helmintex (HTX) methods and point-of-care immunodiagnostic for detecting *Schistosoma* cathodic circulating antigen method (POC-CCA) in samples from Candeal, Estância, Sergipe, northeastern Brazil, November 2015.

Parameters evaluated	KK % (95% CI)	HTX % (95% CI)	POC-CCA t–ve (*) % (95% CI)	POC-CCA t +ve (**) % (95% CI)
**Sensitivity**	29.3 (22.9–36.3)	100.0 (98.1–100.0)	57.4 (50.0–64.6)	81.9 (75.7–87.1)
**Specificity**	100.0 N/A (***)	100.0 (98.7–100.0)	71.1 (65.3–76.4)	35.5 (29.9–41.5)
**PPV**	100.0 N/A	100.0 N/A	57.7 (52.2–63.1)	46.7 (43.9–49.4)
**NPV**	67.2 (65.2–69.2)	100.0 N/A	70.8 (66.9–74.4)	74.0 (66.9–80.1)
**Accuracy**	71.1 (67.0–75.3)	100.0 N/A	65.5 (61.2–69.8)	54.4 (49.9–59.0)
**Prevalence**	11.9 (9.0–14.9)	40.8 (36.3–45.4)	40.6 (36.1–45.0)	71.6 (67.5–75.7)

(*) POC-CCA t–ve refers to the results obtained with the POC-CCA method when the “Trace” results were considered negative

(**) POC CCA t +ve refers to the results obtained with the POC-CCA method when the “Trace” results were considered positive.

(***) N/A, not available.

The urine samples were analyzed with a POC-CCA kit and the results were recorded in two different ways (Tables 2–5). If “trace” results were treated as positive, as recommended by the POC-CCA kit manufacturer, 34 (7.4%) and 8 (1.7%) individuals that were diagnosed as positive for *S*. *mansoni* infection by the HTX and KK methods, respectively, would be incorrectly classified as negative by the POC-CCA method. In contrast, when a comparison was made of the POC-CCA data with the examination of 187 samples with egg detected by HTX, 177 (38.4%) and 283 (61.4%) individuals would have false-positive results, respectively. In the subsets of samples containing < 1 epg or ≥ 1 epg, the POC-CCA method produced the lowest proportion of true-positive results (44% vs. 88%, respectively) and the highest proportion of false-negative results (24% vs. 3%, respectively) (Table 7)

**Table 7:** Distribution of POC-CCA method results according to egg burden (epg <1 and ≥1) in 187 samples collected from Candeal, Estância, Sergipe, northeastern Brazil, November 2015.

Detection result	epg < 1	%	epg ≥ 1	%	Total	%
**Positive**	57	44.2^**a**^	51	87.9^**b**^	108	57.8
**Negative**	31	24.0^**a**^	2	3.4^**b**^	33	17.6
**"Trace"**	41	31.8^**a**^	5	8.6^**b**^	46	24.6
	129	100.0	58	100.0	187	100.0

POC-CCA, point-of-care immunodiagnostic for detecting schistosome cathodic circulating antigen; epg, egg per gram, as estimated by HTX.

^**(a, b)**^ Different letters in the same line indicate a significant difference between the two proportions at 0.01 level of significance by using Fisher Exact Test.

### Values for the estimated prevalence of schistosomiasis in the Candeal community differed widely according to the method used to diagnose the infection

The estimates of schistosomiasis prevalence varied from 11.9% (KK) to 71.6% (POC-CCA, with “trace” considered positive). The prevalences estimated from examining KK-slide1 and KK-slide2 were identical (8.7%), yet were lower than the prevalence estimate that was calculated when the results from slides 1 and 2 were combined (11.9%) (S3 Table). When prevalence estimates were analyzed by the HTX and POC-CCA methods (with the “trace” considered negative), the value was identical (40.6%), but Kappa coefficient was low (0.156) indicating poor agreement between these methods (Table 8).

**Table 8:** A comparison of the positive (1) and negative (0) results obtained when the KK, POC-CCA (CCA) (Trace considered as “positive” or “negative”), and HTX methods were applied to 461 samples were collected in Candeal, Estância, Sergipe, northeastern Brazil, November 2015.

** **	Positive (1) or Negative (0) results		Trace "positive"		Trace "negative"	
KK	CCA	HTX	Sum	%	Sum	%
+	+	+	47	10.2	40	8.7
+	+	-	0	0	0	0
+	-	-	1	0.2	1	0.2
+	-	+	7	1.5	14	3.0
-	-	-	97	21.0	194	42.0
-	-	+	26	5.6	65	14.1
-	+	+	107	23.2	68	14.8
-	+	-	176	38.2	79	17.1
			461	100	461	100

KK, Kato-Katz method; HTX, Helmintex method; POC-CCA, point-of-care immunodiagnostic for detecting schistosome cathodic circulating antigen method.

Kappa coefficients: 0.329 (HTX versus KK); 0.156 (POC-CCA trace positive); 0.285 (POC-CCA trace negative).

### The Candeal community includes a high prevalence of infected individuals with low egg burdens (low infection intensity)

**Fig 2:**
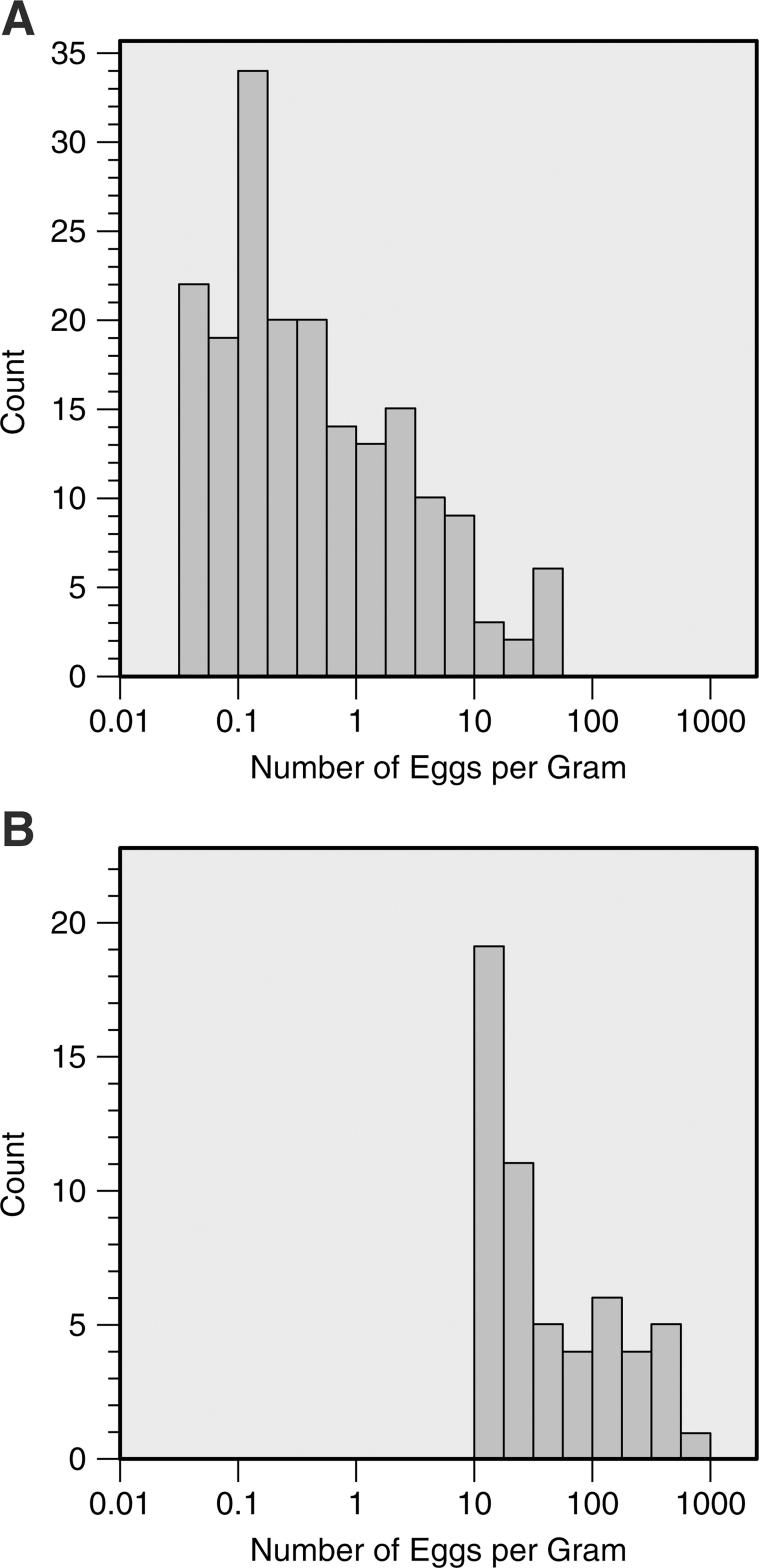
Egg counting by Helmintex (HTX) and Kato-Katz (KK) methods. (A) Distribution of HTX-measured faecal egg burdens in the 187 subjects who tested positive and (B) distribution of the KK-measured faecal egg burdens in the 55 subjects who tested positive. Note the logarithmic scale on the horizontal axis.

The majority of the infected individuals examined presented low egg burdens. For example, among the 187 positive cases diagnosed by the HTX method, 131 (70%) cases involved < 1 epg (Fig 2). In contrast, 56 epg was the highest result obtained with the HTX method. When the epg numbers obtained for the samples with the HTX and KK methods were compared, 46 and 14 samples, respectively, had between 1 and 12 epg, while 23 samples had epg values >50.

### Egg burden is estimated differently by the HTX and KK methods

**Fig 3:**
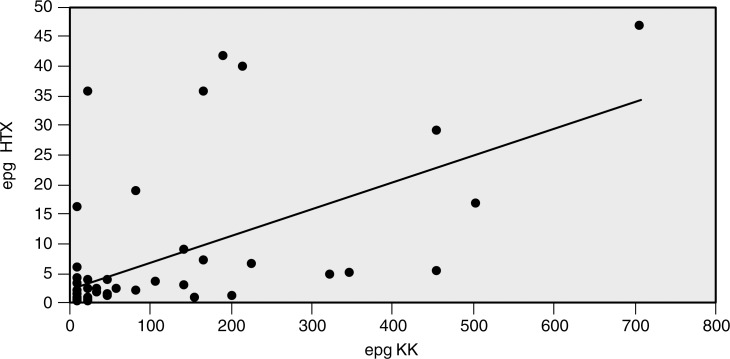
Case-to-case comparisons of the egg burden values estimated by the KK and HTX methods. Correlation coefficient is 0.5615 and 96% of the samples have higher epg values detected by the KK method.

Egg burdens estimated with the KK method were 2.1 to 720 times higher than those estimated with the HTX method in 96% of the samples, resulting in a correlation coefficient of 0.5615 (Fig 3). Meanwhile, for only two samples the HTX method estimated 1.3- and 1.5-times higher epg values than KK (S1 Table).

### POC-CCA band intensity partially correlates with egg burden when epg is equal or higher than 1

The average egg burden for POC-CCA band intensity categories were similar: 0.30 (strong), 0.33 (medium), and 0.26 (weak), in the epg <1 samples subset. Within the epg ≥1 group, there was an increase in the epg average from 3.37 (weak) to 11.61 (medium) and 14.70 (strong). The proportions of samples with strong reactivity have increased from 3.5% (epg<1) to 23.5% (epg≥1), while those with weak reactivity decreased from 71.9% (epg<1) to 39.2% (epg≥1) (Table 9).

**Table 9:** Distribution of POC-CCA method positive results according to egg burden (epg <1 and ≥1) and intensity of the reaction in 108 samples collected from Candeal, Estância, Sergipe, northeastern Brazil, November 2015.

Intensity	epg < 1			epg ≥ 1			Total	%
	n	%	Average		%	Average		
Strong	2	3.5	0.30^**a**^^**A**^	12	23.5	14.70^**b**^ ^**B**^	14	13.0
Medium	14	24.6	0.33^**a**^^**A**^	19	37.3	11.61^**b**^^**A**^^**B**^	33	30.5
Weak	41	71.9	0.26^**a**^^**A**^	20	39.2	3.37^**b**^^**A**^	61	56.5
	57	100.0		51	100.0		108	100.0

POC-CCA, point-of-care immunodiagnostic for detecting schistosome cathodic circulating antigen; epg, egg per gram.

^**(**a,b**)**^ Different letters in the same line indicate a significant difference between the two means at 0.05 level of significance by using t-test (equal variances not assumed).

^**(A, B)**^ Different letters in the same column indicate a significant difference between the means at 0.05 level by using post-hoc Tukey’s test.

### An initial fecal mass less than the standard 30 g mass does not appear to influence HTX performance

Among the 461 fecal samples that were analyzed by the HTX method, only 7 samples had volumes less than the standard 30 g typically analyzed (25 g, 22 g, 20 g, 20 g, 12 g, 12 g, and 12 g). The number of eggs detected in these samples were 5, 4, 90, 3, 10, 24, and 1, respectively; which corresponds to epg estimates of 0.1, 0.13, 3, 0.1, 0.33, 0.8, and 0.03, respectively. In this group of samples, eggs were only detected by the KK method in one sample (the 12 epg sample). These results indicate that the HTX method performs well with fecal masses less than 30 g.

## Discussion

Andrews [25] previously proposed that the sensitivity of egg-detecting methods increases with the volume of biological material that is examined. The HTX method is applied to 30 g of feces and was developed after observing that *S*. *mansoni* eggs could be isolated from feces based on their interactions with paramagnetic particles in a magnetic field [16]. Thus, rather than screening filtered fecal samples, as occurs with the KK method, the eggs that are present in a larger volume of feces can be concentrated into a smaller volume with the HTX method in order to be more easily screened by microscopy. In seeding experiments, HTX method was 100% sensitive with egg burdens higher than 1.3 epg [16]. HTX processing takes approximately 3h and its current estimated cost is US$ 3 per sample (a single KK slide preparation costs US$ 0.2 and POC-CCA costs US$ 1). More recently, the HTX method has been improved by introducing a detergent (Tween 20) at the concentration step, and then staining the final sediment with ninhydrin prior to microscopic evaluation [19]. As a result, significantly less time is spent screening sediment samples, reducing the overall cost of the HTX method. However, it is also recognized that even with recent optimizations of the HTX procedure [19], the HTX method remains labor intensive and not applicable as a routine field diagnostic. Therefore, it is proposed that the HTX method should serve as a reference method for evaluating other methods.

The KK method is operationally simple and inexpensive, and is the diagnostic method recommended by the WHO for epidemiological studies [12]. However, this method lacks sensitivity when fewer eggs are present in a sample [13]. In a number of observational studies where “infected” and “non-infected” individuals were evaluated based on use of the KK method, false negatives probably occurred preventing a correct interpretation of the data [26]. In the present study, the HTX method exhibited higher sensitivity than the KK method. If this is confirmed in future studies, then the HTX method would represent the best method for obtaining a precise determination of infection status by egg detection. This determination is particularly critical for vaccine efficacy evaluations, individual clinical diagnoses, and control of cure efforts, especially in non-endemic countries [26,27,28].

Superior sensitivity of the HTX method compared with KK was previously demonstrated in field-based studies that were conducted in low endemic areas in Brazil [29,30]. In the present survey that was conducted in Candeal, Brazil, the HTX method detected eggs in 29% of the samples that were negative according to the KK method. In addition, the prevalence estimated by the HTX method was 3 times higher than the KK method (40% vs. 11%, respectively) (Table 1). The assessment of relative diagnostic performance by latent class analysis also clearly indicated that the HTX method provided high sensitivity and displayed an overall better performance (Table 6).

Egg burdens are predominantly low among infected individuals in the Candeal community, with 70% of them harboring less than 1 epg. It is noteworthy that low infection intensity is also associated with morbidity and should be targeted in late stages of schistosomiasis elimination [31]. This locality has been under surveillance and regular treatment for many years by the local Ministry of Health authorities. As a result, a high-prevalence, yet low infection-intensity profile has developed in the community. This is in contrast with the more typical coupling of high prevalence and intensity of infections. The potential for prevalence and intensity of infections to be dissociated should be considered in future epidemiological studies and should be used to adjust control measures appropriately. In addition, classification of endemicity needs to account for both prevalence and intensity [3].

With the exception of two samples, egg burdens were found to be higher with the KK method than with the HTX method (Fig 3). While both methods include concentration steps (sieving–KK and HTX; isolation with magnetic particles–HTX; see Fig 1), estimation of epg by the HTX method derives from absolute counting of eggs in 30 g of sample and the KK method estimates epg based on an extrapolation of egg counting in 42 mg of sample. Consequently, the latter potentially contributes to overestimated epg values. This interesting aspect is consistent with discussions in the field regarding the randomness of *S*. *mansoni* egg distribution in feces [14]. With the KK method only examining 42 mg of fecal samples, the possibility that eggs are unevenly distributed would become more evident when lower numbers of eggs are present. This was observed in the present study. It is also possible that HTX underestimates epg because of its estimated egg recovery of aproximately 27% in seeding experiments [19].

POC-CCA is a rapid antigen-based detection test that is applied to urine samples. It has received increasing attention as a promising point-of-care field diagnostic tool, especially based on its use in high endemic areas (e.g., areas with high prevalence and intensity of infections). However, evaluations of this rapid test in low endemicity (specifically low intensity) areas is urgently needed [32,33,34]. The set of samples evaluated in the present study provided an opportunity to directly evaluate the performance of the POC-CCA method with predominantly low intensity infections in comparison with a very sensitive egg detection method (HTX). After LCA analysis, a higher probability of false-positive results is indicated by low positive predictive values (PPV = 46.7% when “trace” is considered positive) (Table 6). Variability in daily egg excretion may explain POC-CCA positive and egg-negative detection and this issue should be addressed in future studies together with appropriate protocol adjustments to minimize cross-contamination in order to avoid false-positive egg detection. Furthermore, performance of the POC-CCA method was worse in the subset of samples that contained less than 1 epg, with a lower detection of “true-positives” and a higher number of “false-positive” results observed (Table 7). Correlation of band intensity and egg burden is also poor, especially with samples containing less than 1 epg (Table 9). Thus, the limitations of the POC-CCA assay for diagnosis of schistosomiasis in individuals that eliminate low numbers of eggs in stool were demonstrated.

In conclusion, the results of the present study support the two initial hypotheses. First, medium-highly endemic areas (defined by prevalence) are suitable for evaluating the diagnostics performance of egg detection methods if a large number of low intensity infections are present, as is the case in Candeal. Thus, “low endemicity areas” with low numbers of positive samples should be avoided when evaluating detection methods. Second, the HTX method is very sensitive and should be used as a reference method for diagnosing intestinal schistosomiasis and for comparative evaluation of other tests. The HTX method should also be considered for use in the monitoring and certification of transmission interruption.

## Supporting information

S1 Checklist The STARD-2015 checklist. (DOCX)

S1 Table Data on 461 samples from Candeal, November 2015. All samples that had feces, urine and serum collected for the study and results from Kato-Katz, Helmintex and POC-CCA.
(XLSX)

S2 Table Data on 187 samples from Candeal, November 2015. The samples where egg was detected by Helmintex and Kato Katz, and the results from POC-CCA examination.
(XLSX)

S3 Table Data on 55 samples from Candeal, November 2015. Evaluation of positivity in each Kato-Katz slide.
(XLSX)

S1 Flow Diagram STARD-2015. (DOCX)
