# Supplementary material for: Study of diagnostic accuracy of Helmintex, Kato-Katz, and POC-CCA methods for diagnosing intestinal schistosomiasis in Candeal, a low intensity transmission area in northeastern Brazil
Source: PLoS Negl Trop Dis. 2018 Mar 8;12(3):e0006274. doi: 10.1371/journal.pntd.0006274 (PMC5843168; doi:10.1371/journal.pntd.0006274)
Supplement: S1 Checklist — (DOCX) [file pntd.0006274.s001.docx]

**S1 Checklist. The STARD 2015 checklist**

| **Section and topic** | **No** | **Item** |
| --- | --- | --- |
| **Title or abstract** | | |
|  | 1 | **Page 1 (Title page)**  Identification as a study of diagnostic accuracy using at least one measure of accuracy (such as sensitivity, specificity, predictive values, or AUC) |
| **Abstract** | | |
|  | 2 | **Page 2**  Structured summary of study design, methods, results, and conclusions (for specific guidance, see STARD for Abstracts) |
| **Introduction** | | |
|  | 3 | **Pages 4-6**  Scientific and clinical background, including the intended use and clinical role of the index test |
|  | 4 | **Page 6, Lines 3-10**  Study objectives and hypotheses |
| **Methods** | | |
| Study design | 5 | **Page 6, Line 14**  Whether data collection was planned before the index test and reference standard were performed (prospective study) or after (retrospective study) |
| Participants | 6 | **Pages 6-7**  Eligibility criteria |
|  | 7 | **Page 6, Lines 14-20**  On what basis potentially eligible participants were identified (such as symptoms, results from previous tests, inclusion in registry) |
|  | 8 | **Page 6, Lines 14-20**  Where and when potentially eligible participants were identified (setting, location, and dates) |
|  | 9 | **Page 6, Lines 14-20**  Whether participants formed a consecutive, random, or convenience series |
| Test methods | 10a | **Pages 7-9**  Index test, in sufficient detail to allow replication |
|  | 10b | **Page 10, Line 16 = no reference standard, but latent class analysis modelling**  Reference standard, in sufficient detail to allow replication |
|  | 11 | **Page 10, Line 16 = no reference standard, but latent class analysis modelling**  Rationale for choosing the reference standard (if alternatives exist) |
|  | 12a | **Page 10, Lines 9-13**  Definition of and rationale for test positivity cut-offs (**No cut-offs were considered**) or result categories of the index test, distinguishing pre-specified from exploratory |
|  | 12b | **Page 10, Line 16 = no reference standard, , but latent class analysis modelling**  Definition of and rationale for test positivity cut-offs or result categories of the reference standard, distinguishing pre-specified from exploratory |
|  | 13a | **Page 10, Lines 9-13**  Whether clinical information and reference standard results were available to the performers or readers of the index test |
|  | 13b | **Page 10, Line 16 = no reference standard, , but latent class analysis modelling**  Whether clinical information and index test results were available to the assessors of the reference standard |
| Analysis | 14 | **Page 10, Lines 15-22**  Methods for estimating or comparing measures of diagnostic accuracy |
|  | 15 | **Page 10, Lines 9-13**  How indeterminate index test or reference standard results were handled |
|  | 16 | **Page 11, Lines 11-13 = only paired results were included in the study**  How missing data on the index test and reference standard were handled |
|  | 17 | **Page, 13 (Table 2)**  Any analyses of variability in diagnostic accuracy, distinguishing pre-specified from exploratory |
|  | 18 | **Page 6, Line 14-20**  Intended sample size and how it was determined |
| **Results** | | |
| Participants | 19 | **See Suplementary file 5**  Flow of participants, using a diagram |
|  | 20 | **Page 11, Lines 11-17**  Baseline demographic and clinical characteristics of participants |
|  | 21a | **Page 6, Lines 20-22**  Distribution of severity of disease in those with the target condition |
|  | 21b | **Page 6, Lines 20-22**  Distribution of alternative diagnoses in those without the target condition |
|  | 22 | **Page 6, Lines 20-22**  Time interval and any clinical interventions between index test and reference standard |
| Test results | 23 | **Pages 11/12 (Table 1) and Page 13 (Table 2)**  Cross tabulation of the index test results (or their distribution) by the results of the reference standard |
|  | 24 | **Page 13 (Table 2)**  Estimates of diagnostic accuracy and their precision (such as 95% confidence intervals) |
|  | 25 | **Page 10, Line 8 = no adverse effects**  Any adverse events from performing the index test or the reference standard |
| **Discussion** | | |
|  | 26 | **Page 21, Line 11-14**  Study limitations, including sources of potential bias, statistical uncertainty, and generalisability |
|  | 27 | **Page 21, Lines 22-25 and Page 22, Lines 1-6**  Implications for practice, including the intended use and clinical role of the index test |
| **Other information** | | |
|  | 28 | **Page 7, Line 6**  Registration number and name of registry |
|  | 29 | **Pages 7-10**  Where the full study protocol can be accessed |
|  | 30 | **In the electronic forms from PLoS**  Sources of funding and other support; role of funders |

*At the start of each item row, authors should specify the page number of the manuscript where the item can be found.
