# Supplementary material for: Study of diagnostic accuracy of Helmintex, Kato-Katz, and POC-CCA methods for diagnosing intestinal schistosomiasis in Candeal, a low intensity transmission area in northeastern Brazil
Source: PLoS Negl Trop Dis. 2018 Mar 8;12(3):e0006274. doi: 10.1371/journal.pntd.0006274 (PMC5843168; doi:10.1371/journal.pntd.0006274)
Supplement: S1 Flow Diagram — (DOCX) [file pntd.0006274.s005.docx]

**S1 STARD Flow Diagram**

Potentially eligible participants

n= 580

Excluded

n= 119

Reason: one of biological samples (feces, urine, serum) was not collected

Eligible participants

n= 461

Index Tests: Kato-Katz (KK), Helmintex (HTX) and POC-CCA (CCA)

n= 461

HTX

negative

n= 274

KK

negative

n= 406

CCA Trace-

negative

n= 275

CCA Trace+

positive

n= 330

CCA Trace+

negative

n= 131

HTX

positive

n= 187

KK

positive

n= 55

CCA Trace-

positive

n= 187

There was no reference standard; comparative evaluation was performed with latent class analysis (LCA)
